# Complete mitochondrial genome of *Rusa unicolor cambojensis* (Artiodactyla: Cervidae)

**DOI:** 10.1080/23802359.2021.1997118

**Published:** 2021-11-10

**Authors:** Guogang Li, Wei Guo, Yunchun Zhang, Guanghong Cao, Zhengli Wang

**Affiliations:** a College of Life Sciences, Qinghai Normal University, Xining, Qinghai, China; b Center for Integrative Conservation, Xishuangbanna Tropical Botanical Garden, Chinese Academy of Sciences, Mengla, Yunnan, China; c Academy of Plateau Science and Sustainability, Qinghai Normal University, Xining, Qinghai, China; d Linnause Labs Technology Co., Ltd, Wuyuan, Jiangxi, China; e State Key Laboratory of Genetic Resources and Evolution, Yunnan Laboratory of Molecular Biology of Domestic Animals, Kunming Institute of Zoology, Chinese Academy of Sciences, Kunming, Yunnan, China; f Naban River Watershed National Nature Reserve, Jinghong, Yunnan, China

**Keywords:** Mitogenome, phylogenetics, *Rusa unicolor cambojensis*

## Abstract

*Rusa unicolor* has been listed as a vulnerable species by the International Union for Conservation of Nature and Natural Resources because of human activities. In recent years, population numbers have decreased due to heavy hunting and habitat loss, and little genetic data on this species exists; thus, our knowledge of range distribution and population size remains limited. In the current study, the complete *R. u. cambojensis* mitochondrial genome was sequenced using polymerase chain reaction followed by direct sequencing. The complete mitochondrial genome was determined to be circular and contain 16,557** **bp, including 13 protein-coding genes, 2 rRNA genes, 22 tRNA genes, and 1 control region, the gene composition and order were similar to those of most other vertebrates reported to date. Most mitochondrial genes, except for *ND6* and eight tRNAs, were encoded on the heavy strand. The overall base composition of the heavy strand was 33.6% A, 28.9% T, 24.2% C, and 13.3% G, with a strong AT bias of 62.5%. There were 13 regions of gene overlap totaling 96** **bp and 12 intergenic spacer regions totaling 70** **bp. The phylogenetic analyses (maximum likelihood and Bayesian inference) of *R. unicolor* based on the mitochondrial genome four subspecies of *R. unicolor* were clustered into a well-supported single clade, and *R. u. cambojensis* was most closely related to *R. u. dejeani*. This study will assist in the exploration of the evolutionary history and taxonomic status of the sambar, as well as its protection as a genetic resource.

*Rusa unicolor* (Kerr 1792, Artiodactyla: Cervidae) or the sambar is the most widespread deer in Asia (Pocock 1943; Grubb 1990; Leslie 2011). It is listed as vulnerable on the International Union for Conservation of Nature and Natural Resources Red List because of its reduced range (Timmins et al. 2015). Characteristics of single genes (e.g. mitochondrial *16S rRNA, Cytb*, and *COI*) have been used to distinguish *R. unicolor* from other species (Chen et al. 1991; Liu et al. 2003; Guha and Kashyap 2005; El-Jaafari et al. 2008; Kumar et al. 2012; Cai et al. 2016). The mitochondrial genome is better for investigating species evolution and population genetics (Hassanin et al. 2012). Within the seven subspecies, only the genomes of *R. u. swinhoei*, *R. u. dejeani*, and *R. u. hainana* are available (Chen et al. 2011; Wu et al. 2016; Liu et al. 2019). In the current investigation, the complete mitochondrial genome of *R. u. cambojensis* was sequenced and characterized to provide fundamental molecular data for further conservation and phylogenetic studies of this large mammal. Possible relationships between subspecies of *R. unicolor* are also discussed.

One dead *R. u. cambojensis* specimen was collected from Naban River Watershed National Nature Reserve (100°42′ E, 22°14′ N), Xishuangbanna, Yunnan Province, southwestern China on 8^th^ July 2017. The sample (1707001) was deposited in the Center for Integrative Conservation, Xishuangbanna Tropical Botanical Garden, Chinese Academy of Sciences, Mengla, Yunnan province, China. Total genomic DNA was extracted from tissue using a DNeasy Blood & Tissue Kit (Tiangen Biochemistry Technology Co., Ltd., China) and sequenced using an ABI PRISM 3700 sequencing system (Applied Biosystems, Foster City, CA, USA). To obtain the whole mitogenomic sequence, 22 pairs of primers were designed according to previous studies (Chen et al. 2011; Hassanin et al. 2012; Wu et al. 2016; Liu et al. 2019). The base composition of the mitochondrial genome was analyzed using MEGA 5.05 (Tamura et al. 2011). The genome sequence was annotated using DOGMA (Wyman et al. 2004) and was deposited on the NCBI website (https://www.ncbi.nlm.nih.gov/genbank/) with the accession number MK941883. Features of the complete mitochondrial genome of *R. u. cambojensis* were identical to those of other sambar subspecies (e.g. Chen et al. 2011; Wu et al. 2016; Liu et al. 2019). It was a circular molecule, 16,557** **bp in length, and included 13 protein-coding genes, 2 rRNA genes, 22 tRNA genes, and 1 non-coding control region (D-loop). The overall base composition of the heavy strand was 33.6% A, 28.9% T, 24.2% C, and 13.3% G, with a strong AT bias of 62.5%.

Phylogenies of the mitogenome were constructed using maximum likelihood, implemented in PHYML 3.0 (Guindon et al. 2010). Bayesian inference was implemented in MRBAYES 3.2.1 (Ronquist et al. 2012). Based on the complete genomes (Figure 1), four subspecies of *R. unicolor* were clustered in a well-supported single clade, with *R. timorensis* as its sister species. Intraspecific phylogenetics demonstrated that *R. u. cambojensis* was more closely related to *R. u. dejeani* than to *R. u. swinhoei* and *R. u. hainana*, which are respectively endemic to the islands of Taiwan and Hainan. The *R. u. cambojensis* mitogenome will be useful for its identification and conservation, as well as for evolutionary research on *R. unicolor*.

**Figure 1.**
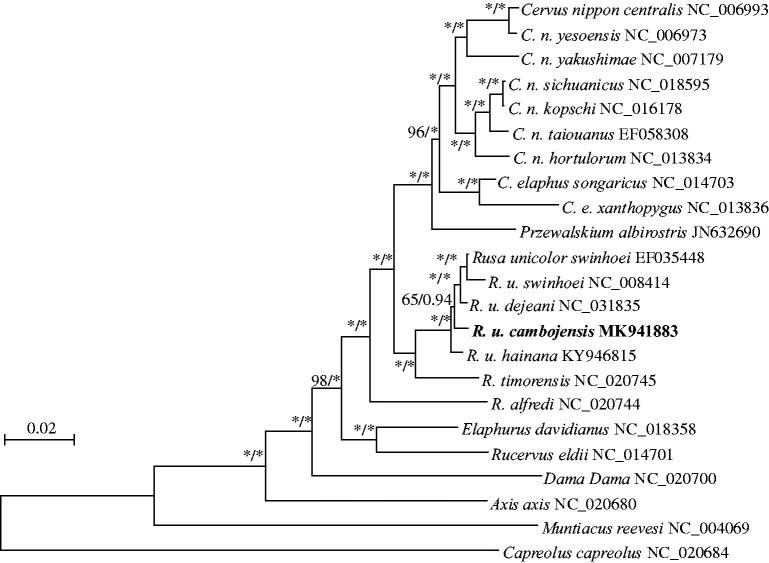
Maximum likelihood and Bayesian inference phylogenetic trees for *Rusa unicolor* based on 23 complete mitochondrial genomes. Numbers on branches indicate bootstrap support for maximum likelihood, followed by the posterior probability in Bayesian inference analyses for the node. Stars indicate values of 100 for maximum likelihood and 1.00 for Bayesian inference.

## Data Availability

The datasets supporting the results of this article are available in GenBank of the NCBI at (https://www.ncbi.nlm.nih.gov/) under accession number MK941883.
